# Registro de Intervenções Coronárias em Vasos de Muito Pequeno Calibre: Análise de Stent Eluidor de Sirolimus e Outros Stents Contemporâneos

**DOI:** 10.36660/abc.20250145

**Published:** 2026-02-27

**Authors:** João Vitor Slaviero, André Luiz Langer Manica, Rogério Eduardo Gomes Sarmento Leite, Eduarda Paiva Borsa, Marcia Moura Schmidt, Rodrigo Campos Ogando, Julia Kurtz Teixeira, Raphael Boesche Guimarães, Alexandre Damiani Azmus, Carlos A. M. Gottschall

**Affiliations:** 1 Instituto de Cardiologia Porto Alegre RS Brasil Instituto de Cardiologia, Porto Alegre, RS – Brasil; 2 Universidade Federal de Ciências da Saúde de Porto Alegre Porto Alegre RS Brasil Universidade Federal de Ciências da Saúde de Porto Alegre, Porto Alegre, RS – Brasil; 3 Pontifícia Universidade Católica do Rio Grande do Sul Porto Alegre RS Brasil Pontifícia Universidade Católica do Rio Grande do Sul, Porto Alegre, RS – Brasil

**Keywords:** Stents Farmacológicos, Sirolimo, Intervenção Coronária Percutânea, Doença da Artéria Coronariana

## Abstract

**Fundamento:**

A intervenção coronária percutânea (ICP) de vasos de muito pequeno calibre (≤ 2,25mm) é um desafio técnico e tem piores resultados quando comparado aos maiores calibres. O stent Inspiron^®^ é um stent farmacológico (SF) feito no Brasil, o qual têm segurança e eficácia avaliadas por diversos estudos em diferentes cenários, mas não há dados analisando seu desempenho em vasos <2,5mm.

**Objetivos:**

Analisar os resultados clínicos de ICP com SF’s em vasos de muito pequeno calibre e comparar com outras plataformas de SF’s contemporâneos. O desfecho MACE foi definido como por mortalidade cardíaca, infarto da lesão alvo e revascularização de lesão alvo clinicamente dirigida.

**Métodos:**

Estudo observacional, com amostra consecutiva em centro de referência no Brasil, entre 2017 e 2021, tendo desfechos analisados nos períodos intra-hospitalar, 30 dias, seis e 12 meses, com nível de significância estatística de 5%.

**Resultados:**

Um total de 783 SF’s foram implantados, sendo 47% INSPIRON^®^ e 46,8% “Outros SF’s”. A idade média dos pacientes foi de 64,7±11 anos, 61% dos pacientes masculinos e 42% portadores de Diabetes Mellitus. Aos 12 meses, o desfecho de MACE geral foi 4,54% (36/793) e foi similar entre os grupos comparados (Inspiron^®^-4,6%; *vs.* . “Outros SF’s” -4,6%; p=1,000), sem diferença significativa nos desfechos individuais. A taxa de trombose de stent foi similar entre os grupos (4 [1,0%] *vs.* 2 [0,5%], p 0,868).

**Conclusão:**

Nossos achados demonstram resultados aceitáveis e com desempenho similar em 12 meses do Inspiron^®^ a outros SF’s contemporâneos em vasos de muito pequeno calibre.

## Graphical abstract

**Figura Central:**
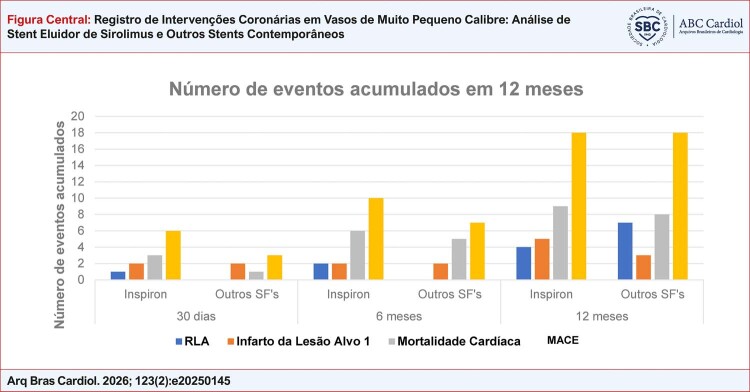
Registro de Intervenções Coronárias em Vasos de Muito Pequeno Calibre: Análise de Stent Eluidor de Sirolimus e Outros Stents Contemporâneos

RLA: Revascularização de lesão alvo; Mortalidade Cardíaca: Mortalidade cardíaca confirmada e provável; MACE: mortalidade cardíaca, infarto da lesão alvo e revascularização de lesão alvo clinicamente dirigida.

## Introducão

A intervenção coronária percutânea (ICP) vem aumentando sua complexidade como consequência da melhora na tecnologia dos materiais, da expertise dos operadores e das técnicas dos procedimentos.^1 , 2^ Nos pacientes submetidos a ICP, a prevalência de doença de pequenos vasos (<2,75mm) tem sido relatada em até 50% dos casos.^3 - 5^ Com a subsequente disponibilidade de stents de 2,0 e 2,25 mm, o termo “vaso de muito pequeno calibre” foi proposto.^6^

A ICP de vasos de pequeno calibre constitui um desafio estando associada a maior risco de eventos cardíacos, incluindo reestenose e trombose de stent.^4 , 7 - 16^ Ademais, estas características anatômicas geralmente acompanham um padrão de doença coronariana aterosclerótica difusa, necessitando stents de maiores comprimentos, que apresenta relação direta com o risco de reestenose,^13^ além de ser características que desfavorecem o tratamento cirúrgico alternativo.^14 - 16^

Visto o grande número de opções de stent farmacológico (SF’s) disponíveis atualmente, se buscou identificar características que pudessem predizer melhores resultados. Visto que a menor espessura das hastes, pode estar associada a menor trombogenicidade e menor tensão de cisalhamento,^17 - 19^ dados de metanálise comparando espessuras de hastes de SF’s contemporâneos demonstram menores riscos relativos de desenvolvimento de trombose de stent, infarto e revascularização de lesão alvo em SF de hastes menores, mas sem significância estatística. Outra característica explorada foi o fármaco utilizado nos SF’s. A utilização de SF’s parece ter um efeito de classe quanto ao desempenho em vasos de pequeno calibre,^20^ embora em contexto de lesões mais longas os dados de revascularização de lesão alvo e infarto tendem a ser melhores quando utilizados SF’s eluídos com Sirolimus.^21^

O stent INSPIRON^®^ foi o primeiro SF produzido no Brasil e é eluidor de Sirolimus, com liga de cobalto-cromo e haste de 75 μm de espessura, polímero abluminal biodegradável, apresentando a possibilidade de implante em vasos de muito pequeno calibre. Vários estudos clínicos testaram sua segurança e eficácia,^17 , 18 , 22^ mas não há dados clínicos comparando seu desempenho em vasos de calibre ≤2,25mm com outros SF contemporâneos.

Visto o grande número de ICP’s nestes diâmetros e o uso amplamente difundido do Inspiron^®^, nosso objetivo é analisar os resultados de mundo real de ICP’s com SF’s contemporâneos em vasos de muito pequeno calibre.

## Métodos

### Desenho do estudo, local e população

Estudo observacional, prospectivo, por iniciativa de autores, com uma amostra consecutiva de pacientes acima de 18 anos encaminhados ao laboratório de hemodinâmica de hospital terciário de referência no Sul do Brasil, entre 2017 e 2021, com indicação e submetidos a ICP de vasos de muito pequeno calibre com INSPIRON^**®**^ ou outros SF’s contemporâneos. A indicação da intervenção ficou a critério da equipe assistente responsável pelo caso. Não foram incluídos pacientes com stents implantados em lesões coronárias de outros calibres (≥2,5mm) e ICP sem implante de SF’s.

Os dados foram registrados, sendo a coleta feita a partir da revisão de prontuário, consultas presenciais e contato telefônico. Foram coletados os desfechos clínicos intra-hospitalares, em 30 (trinta) dias, seis (06) meses e em 12 (doze) meses. Novas cineangiocoronariografias foram realizadas apenas em pacientes com indicação clínica de estratificação invasiva pela equipe assistente, não sendo realizadas rotineiramente durante o seguimento, assim como testes funcionais.

O presente estudo foi aprovado pelo Comitê Institucional de Pesquisa e Ética da instituição, estando em conformidade com a Resolução CNS 466/2012.

### Aspectos do procedimento

Todos os pacientes realizaram a ICP recebendo ou sendo administrado imediatamente após, dupla antiagregação plaquetária, e heparina não fracionada durante o procedimento. A estratégia técnica do procedimento, o tipo e a duração da terapia antiplaquetária dupla foram definidos pela equipe assistente. Com exceção dos casos que utilizaram imagem intravascular, as demais ICP’s utilizaram a estimativa angiográfica visual para determinação do diâmetro de referência do vaso.

### Stents

Todos os pacientes incluídos receberam pelo menos um SF INSPIRON^**®**^ e/ou outro(s) SF’s. A decisão de implantar o tipo de stent foi baseada no critério do operador e na disponibilidade momentânea no centro médico. As características dos stents utilizados são apresentadas na Tabela 1 .

**Tabela 1:** – Stents utilizados

Stent	N (%)	Material	Espessura	Droga	Polímero
**Inspiron** ^**®**^	392 (50,1%)	Cromo-Cobalto	75μm	Sirolimus	Biodegradável
**Resolute Onyx/ Integrity** ^**®**^	218 (27,8%)	Cromo- Cobalto	81/91μm	Zotarolimus	Durável
**Promus** ^**®**^	73 (9,7%)	Cromo-Platina	81μm	Everolimus	Biodegradável
**Xience** ^**®**^	37 (4,7%)	Cromo- Cobalto	81μm	Everolimus	Durável
**Synergy** ^**®**^	30 (3,8%)	Cromo-Platina	74μm	Everolimus	Biodegradável
**Orsiro** ^**®**^	19 (2,4%)	Cromo- Cobalto	60μm	Sirolimus	Biodegradável
**Ultimaster** ^**®**^	8 (1,0%)	Cromo- Cobalto	80μm	Sirolimus	Biodegradável
**Supraflex** ^**®**^	6 (0,8%)	Cromo- Cobalto	60	Sirolimus	Biodegradável

Tabela referente a stents farmacológicos analisados na amostra, apresentados em ordem de frequência, material de sua haste metálica, espessura de haste metálica, droga eluidora e polímero utilizado.

### Definição de objetivos e desfechos

O desfecho principal MACE ( *Major Adverse Cardiovascular Events* ) foi composto por “Mortalidade Cardíaca”, “Infarto da Lesão Alvo” e “Revascularização de Lesão Alvo Clinicamente Dirigida”. Ademais, foram analisados os componentes individuais do desfecho principal, assim como o desfecho de “Trombose de Stent”, o qual foi definido como infarto agudo do miocárdio associado a oclusão do SF’s 2,00 ou 2,25mm previamente implantando, o que é classificado como “Trombose de stent definida ou confirmada”.^20^

O objetivo do estudo foi analisar o desfecho composto geral da amostra e compará-lo entre os stents INSPIRON^®^ e outros SF’s contemporâneos disponíveis no centro durante o seguimento de um ano. Foi comparada a diferença nas taxas de MACE entre diferentes espessuras de haste (<80μm *vs.* >80 μm), o tipo de droga eluída (sirolimus *vs.* outras drogas).

O sucesso angiográfico da ICP foi definido como uma lesão residual mínima do diâmetro da estenose de <20%, com fluxo TIMI III, sem oclusão de ramo lateral, sem dissecção com comprometimento do fluxo coronário anterógrado, embolização distal ou trombo angiográfico.^21^

### Análise estatística

A análise dos dados foi realizada no sistema *IBM Statistical Package for the Social Sciences* (versão 20.0). Os dados categóricos são apresentados como números (n) e porcentagens (%). As variáveis contínuas são apresentadas como média ± desvio padrão. A normalidade da distribuição de cada variável contínua foi avaliada pelo teste de Kolmogorov-Smirnov. A associação entre variáveis categóricas foi realizada através do teste de Qui-quadrado e se testaram diferenças estatisticamente significativas entre variáveis contínuas pelo teste T de Student não pareado, todos com um nível de significância bilateral de 0,05. As curvas de sobrevida foram estimadas pelo método de Kaplan-Meier, e as diferenças entre os grupos foram comparadas por meio do teste log-rank, adotando-se um nível de significância de 5%.

### Poder do estudo

Com a amostra disponível para realizar o estudo, 391 e 392 pacientes nos grupos avaliados, conseguimos detectar diferenças de aproximadamente 6 pontos percentuais com um poder acima de 80%. Estes cálculos foram realizados através do programa WINPEPI 11.65.

## Resultados

**Figura 1:**
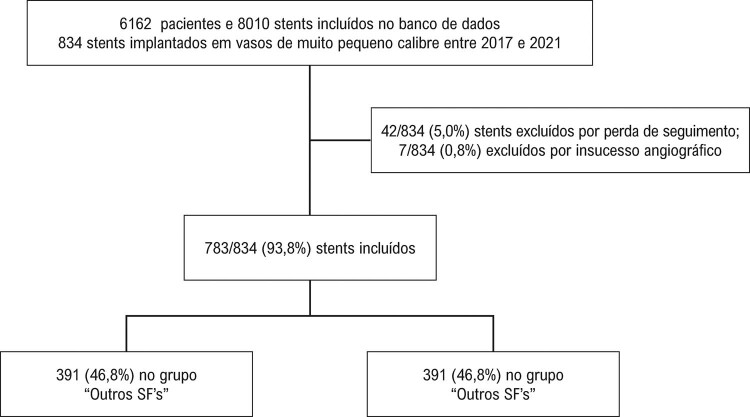
– Fluxograma.

De janeiro de 2017 a dezembro de 2021, 834 stents foram implantados e incluídos no banco de dados, dos quais 783 (93,8%) stents foram implantados com sucesso, preencheram os critérios de inclusão e tiveram seguimento por pelo menos 12 meses, sendo 392 stents INSPIRON^®^ (47%) e 391 “Outros SF’s” (46,8%). Esses 834 stents foram implantados em 762 pacientes (1,03 stent por paciente). Sete stents (0,8%) não foram incluídos no seguimento (quatro no grupo INSPIRON^®^ e três no grupo “Outros SF’s”, *1,02% vs. 0,76%, p 0,760* ) devido a quatro eventos de no-reflow, duas falhas na entrega dos stents devido a tortuosidade e calcificação severas proximais as lesões, e um óbito por fibrilação ventricular durante a liberação do stent ( Figura 1 ).

As taxas de hipertensão, diabetes mellitus, tabagismo ativo e dislipidemia foram semelhantes entre os grupos. A média de idade foi maior no grupo “Outros SF’s” com significância estatística, embora a pequena diferença numérica não tenha uma relevância no impacto clínico dos desfechos. O diagnóstico prévio de insuficiência cardíaca foi maior no grupo “INSPIRON^®^” e a apresentação clínica que motivou a ICP foi por síndrome coronariana aguda em 74,7% das intervenções, das quais o diagnóstico mais frequente foi de angina instável (40,4%), tendo taxa significativamente maior no grupo “Outros SF’s”. Demais informações clinicas são apresentadas na Tabela 2 .

**Tabela 2:** – Características clínicas e demográficas

Características Clínicas e Demográficas	Stents (n = 783)	INSPIRON^**®**^ (n = 392; 50%*)	Outros SF (n = 391; 50%*)	p
Idade em anos	64,7	63,3 ±10,98	66,13±11,15	0,001
IMC kg/ m^2^	27,5	27,26 ±3,55	27,64 ±3,73	0,138
Masculino	480(61,3%)	227(47,2%)	253(52,8%)	0,060
Creatinina (g/dL)	1,04	1,02 ±0,38	1,06±0,40	0,249
HAS	603(77%)	302 (50%)	301 (50%)	1,000
Histórico Familiar de DAC	69 (8,8%)	37 (59,6%)	32 (46,4%)	0,622
Tabagista ativo	293(37,4%)	156 (53,3%)	137 (46,7%)	0,193
DM	329 (42%)	177(53,7%)	152 (46,3%)	0,088
DM em uso de insulina	92 (11,7%)	55 (59,7%)	37(40,3%)	0,091
Dislipidemia	259(33,1%)	121 (46,7%)	138 (53,3%)	0,105
IC – FEVE <50%	48 (6,1%)	33 (68,7%)	15 (31,3%)	0,012
AVE prévio	27 (3,4%)	15 (55,5%)	12 (44,5%)	0,700
Infarto do Miocárdio prévio	121(15,5%)	64 (52,8%)	57(47,2%)	0,563
ICP prévia	162(20,7%)	73 (45%)	89 (55%)	0,180
CRM prévia	64 (8,2%)	34 (53,2%)	30 (46,8%)	0,703

Os stents INSPIRON^®^ foram mais implantados nas artérias circunflexa, coronária direita e artéria descendente posterior em relação ao grupo “Outros SF’s”. Não houve diferença estatística entre os grupos em relação ao comprimento dos stents utilizados, nas vias de acessos arteriais e na porcentagem de stents submetidos a pré-dilatação. As ICP’s com stents INSPIRON^®^ tiveram taxas estatisticamente significativas menores no uso de aterectomia rotacional e ultrassonografia intravascular (intravascular ultrasound – IVUS), no entanto, a taxa de utilização desses dispositivos foi muito baixa, não permitindo fazer correlações com eventos. Demais características angiográficas e do procedimento são apresentadas na Tabela 3 .

**Tabela 3:** – Características angiográficas e do procedimento

Variável	Total n (%)	INSPIRON^**®**^ n (%*)	Outros SF n (%*)	p
**Vaso Coronário Tratado**	**(n = 783)**	(n = 392; 50%*)	(n = 391; 50%*)	
Descendente Anterior	293 (36%)	157 (53,5%)	136 (46,4%)	0,127
Circunflexa	139 (18%)	87 (62,5%)	52 (37,4%)	**0,001**
Coronária Direita	171 (22%)	102 (59,6%)	69 (40,3%)	**0,004**
Ramo Diagonal	68 (8,6%)	27 (39,7%)	33 (60,2%)	0,420
Obtuso Marginal	70 (8,9 %)	41 (58,5%)	29 (41,4%)	0,132
Descendente Posterior	31 (3,9%)	22 (70,9%)	8 (29%)	**0,009**
Póstero-lateral	14 (1,7%)	8 (57,1%)	6 (42,8%)	0,546
Enxerto Venoso	7 (0,89%)	1 (14,3%)	6 (85,7%)	0,057
**Apresentação Clínica**				
Infarto com supra ST	145(18,5%)	81 (55,8%)	64 (44,1%)	0,117
Infarto sem supra ST	124(15,8%)	74 (59,6%)	50 (40,3%)	0,180
Angina Instável	316(40,4%)	127 (40,1%)	189 (59,8%)	**0,001**
Angina Estável	198(25,3%)	110 (55,5%)	88 (44,4%)	0,070
**Avaliação Fisiológica**				
Fisiologia Invasiva	7 (0,89%)	1 (14,2%)	6 (85,7%)	0,057
Cintilografia Miocárdica	18 (2,3%)	6 (33,3%)	12 (66,6%)	0,152
Ecografia com Stress	1 (0,1%)	1 (100%)	0 (0%)	0,320
Teste Ergométrico	11 (1,4%)	5 (45,4%)	6 (54,5%)	0,860
**Aspectos do Procedimento**				
Pré-dilatação	710(90%)	355 (50%)	355 (50%)	0,981
Aspiração de trombos	6 (0,8%)	4 (66,6%)	2 (33,3%)	0,412
Aterectomia Rotacional	9 (1,1%)	0 (0,0%)	9 (100%)	**0,002**
IVUS	8 (1,0%)	0 (0,0%)	8 (100%)	**0,004**
Pós-dilatação	369(47,1%)	171 (46,3%)	198 (53,6%)	**0,049**
Acesso arterial radial	588(75,1%)	285 (48,4%)	303 (51,5%)	0,121
Acesso arterial femoral	195(24,9%)	98 (50,2%)	97 (49,7%)	0,950
Comprimento de stent	21,46	22,05±6,0mm	21,45±7,2mm	0,717
**Diâmetro de stent**				
- 2.0mm	16 (2,0%)	4 (25%)	12 (75%)	0,068
- 2.25mm	767 (97,9%)	386 (50,3%)	381 (49,6%)	0,862

* Proporções referentes às variáveis analisadas separadamente e não ao total da amostra. SF: stent farmacológico; IVUS: intravascular ultrasound – ultrassonografia intravascular.

**Figura 2:**
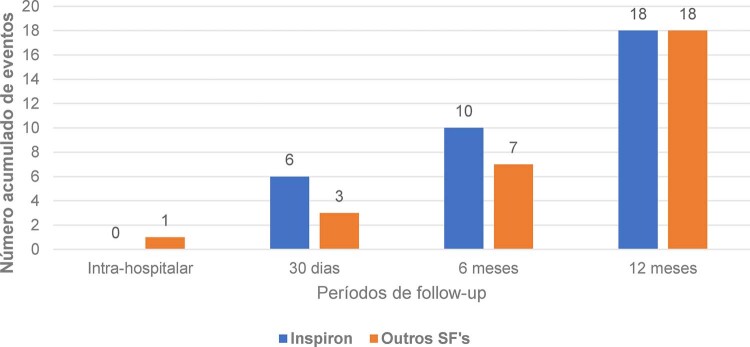
– Eventos de MACE acumulados por períodos avaliados. Número acumulado de eventos de MACE (mortalidade cardíaca, infarto da lesão alvo e revascularização de lesão alvo clinicamente dirigida) durante os períodos de avaliação em cada grupo de stent.

**Figura 3:**
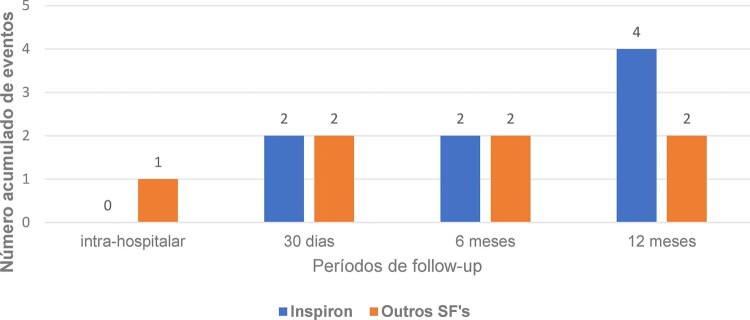
– Trombose de stent durante períodos avaliado. Número acumulado de eventos de trombose de stent durante os períodos de avaliação em cada grupo de stent.

O desfecho MACE geral foi 4,54% (36/793) e foi equivalente estatisticamente entre os grupos comparados ( Tabela 4 ): 18 eventos no grupo “INSPIRON®” e 18 eventos no grupo “Outros SF’s” ( Figura 2 ), assim como cada componente individual do desfecho composto. A taxa de trombose de stent foi similar entre os grupos de stents durante todos os períodos avaliados ( Figura 3 ).

**Tabela 4:** – Desfechos clínicos no acompanhamento de 12 meses

Período Grupo Stent Número de stents	Intra-hospitalar		30 dias		6 meses		1 ano	
	Inspiron^**®**^ 392	Outros 391	Inspiron^**®**^ 392	Outros 391	Inspiron^**®**^ 392	Outros 391	Inspiron^**®**^ 392	Outros 391
**MACE**	0 (0%)	1(0,3%)	6(1,5%)	3 (0,8%)	10 (2,5%)	7(1,8%)	18(4,6%)	18(4,6%)
**“p”**	**p 0,499**		**p 0,505**		**p 0,625**		**p 1,000**	
**RLA**	0 (0%)	0 (0%)	1 (0,2%)	0 (0%)	2 (0,5%)	0 (0%)	4 (1,0%)	7 (1,8%)
**“p”**	**p 1,000**		**p 0,999**		**p 0,499**		**p 0,384**	
**Infarto de lesão alvo**	0 (0%)	1 (0,3%)	2(0,5%)	2(0,5%)	2(0,5%)	2(0,5%)	5(1,3%)	3(0,8%)
**“p”**	**p 0,499**		**p 1,000**		**p 1,000**		**p 0,725**	
Mortalidade cardíaca*	0 (0%)	0(0%)	3 (0,8%)	1 (0,3%)	6 (1,5%)	5(1,3%)	9(2,3%)	8(2,1%)
**“p”**	**p 1,000**		**p 0,624**		**p 0,999**		**p 0,999**	

MACE: Major Adverse Cardiovascular Events (mortalidade cardíaca, infarto da lesão alvo e revascularização de lesão alvo clinicamente dirigida; RLA: revascularização de lesão alvo clinicamente dirigida; Mortalidade cardíaca*: mortalidade cardíaca confirmada e provável.

A taxa de mortalidade cardíaca confirmada e provável total na amostra foi de 2,14% (17/793) similar entre os grupos estudados. O seguimento clínico foi similar entre os grupos, sendo que houve perda de seguimento de 42 pacientes (5,5%) durante o período de um ano.

**Figura 4:**
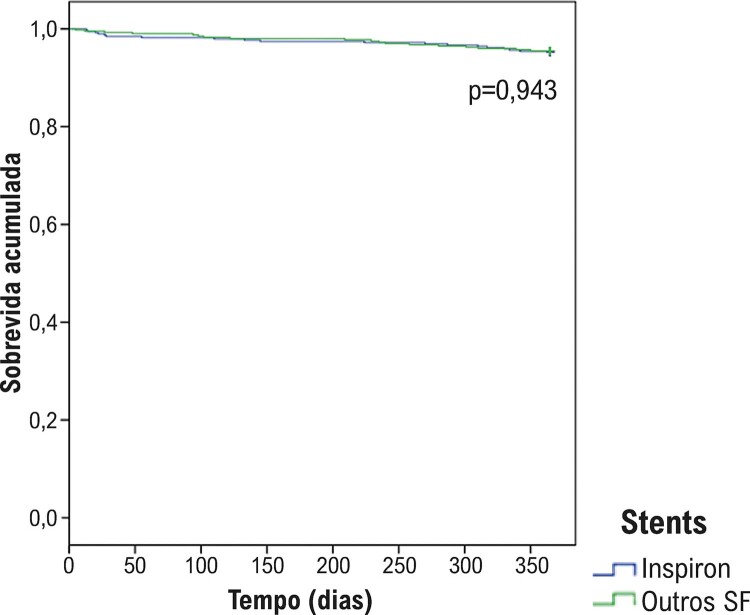
– Curva de Kaplan-Meier. Curva de sobrevida acumulada livre de MACE (mortalidade cardíaca, infarto da lesão alvo e revascularização de lesão alvo clinicamente dirigida) durante o período de 365 dias de seguimento.

Durante o seguimento de um ano, não houve diferença na taxa de stents livres de desfechos ( Figura 4 ). Durante os seguimentos de seis meses e um ano, os grupos mantiveram uso de terapias antiagregantes de forma semelhantes.

## Discussão

Esse registro contemporâneo de mundo real, o qual avaliou os dados clínicos brasileiros em pacientes submetidos a ICP com stent em vasos de muito pequeno calibre, demonstrou baixas taxas de MACE, resultados similares entre os diferentes tipos de dispositivos disponíveis comparados ( Figura Central ) e dados da literatura.^23 , 24^

A presença de diabetes mellitus está frequentemente associada a doença aterosclerótica coronariana difusa em pequenos vasos, além de ser considerada preditor de reestenose e trombose de stent após ICP com SFs.^25 - 35^ Em nossa amostra, dos stents que apresentaram MACE, 42% (15/36) foram implantados em pacientes com diagnóstico prévio de diabetes mellitus, tendo a mesma frequência na amostra geral. A maioria dos pacientes submetidos a ICP necessitaram comprimentos de malha de stents (>20mm), embora os stents com MACE não tiveram comprimento médio maior que os stents livres de eventos (21,98mm *vs.* 21,43mm, p,0820). Ademais, embora algumas diferentes taxas entre os grupos de stents implantados nos aspectos clínicos, a análise de MACE dos stents “INSPIRON^®^” e “Outros SF’s” não demonstrou diferença significativa em pacientes com insuficiência cardíaca com fração de ejeção <50% (4/18 stents [22%] vs. 3 stents [17%], p 0,67), apresentação clínica da intervenção índice como síndrome coronariana aguda (8/18 stents [44,4%] vs. 9/18 stents [50%], p 0,74). O pequeno número de desfechos durante o seguimento, impossibilitou uma análise multivariada de outras características clínicas e do procedimento.

O potencial efeito de menor lesão endotelial pelas plataformas com hastes mais finas, foi testado em nossa amostra quando comparamos stents com hastes <80 µm (447 stents – 57%) e os demais, embora não apresentem significância estatística entre o Inspiron^®^ e Outros SFs (48% vs. 52%, *p=1,010,* respectivamente); É possível que em uma amostra maior de pacientes ou em estudos com acompanhamento angiográfico de rotina seja possível identificar esta diferença uma vez que o desenvolvimento de sintomas agudos ou crônicos, principais motivos para uma nova estratificação invasiva em nossa amostra, pode ser subestimada devido a menor área isquêmica em risco e, consequentemente, menor possibilidade de sintomas.

Ao analisar resultados conforme o tipo de droga eluida nos dispositivos, os dados desta amostra demonstram uma equivalência estatística de MACE entre o stent eluidor de Sirolimus (Inspiron^®^) e dispositivos com outros fármacos, sugerindo haver um efeito de classe dos SF’s disponíveis e corroborando os dados científicos atuais.^36 - 39^ Estes achados, sugerem também que diversas outras características de engenharia e bioquímicas dos SF’s contemporâneos podem minimizar a importância do tipo de droga presente na plataforma.

O uso de imagem intravascular, representado pelo IVUS, impacta em resultados angiográficos e clínicos agudos e a longo prazo.^40 - 43^ A taxa de uso destas ferramentas foi baixa em nossa amostra, apenas 1% (8/783) dos casos, visto que os procedimentos foram realizados, em sua grande maioria, em pacientes da rede pública de saúde, a qual não financiava a tecnologia. Ademais, o uso de imagem intravascular em vasos <2,25 mm pode ser desafiador, aumentando o risco de lesão iatrogênica da placa,^41^ o que pode ter influenciado na escolha do operador.

O padrão de dispersão difusa dos eventos de trombose de stent em nossa amostra sugere que fatores técnicos agudos relacionados ao procedimento, como má expansão e dissecções coronárias significativas, não foram fatores que tiveram alta influência nas taxas de desfechos ( Tabela 5 ).

**Tabela 5:** – Trombose de stent

Período Tipo de Stent N, (%)	Intra-hospitalar		30 dias		6 meses		1 ano	
	Inspiron^**®**^	Outros	Inspiron^**®**^	Outros	Inspiron^**®**^	Outros	Inspiron^**®**^	Outros
Trombose de stent*	0 (0%)	1 (0,3%)	2 (0,5%)	2(0,5%)	2(0,5%)	2 (0,5%)	4(1,0%)	2(0,5%)
**“p”**	**p 0,499**		**p 1,000**		**p 1,000**		**p 0,868**	

Taxa cumulativa de trombose de stent durante períodos. Trombose de stent*: Trombose de stent confirmada.

O uso e duração da terapia antiplaquetária após ICP foram similares durante o seguimento dos pacientes, com alta taxa manutenção de dupla antiagregação aos 12 meses, o que pode ter influenciado na frequência de desfechos ( Tabela 6 ).

**Tabela 6:** – Terapia médica antiplaquetária

Terapia Médica aos 6 meses, n* (%)	INSPIRON^**®**^ (382/ 392)	Outros SF (383/ 391)	“p”
AAS	364 (95,2%)	369 (96,3%)	0,242
**Inibidores da ADP**			
Clopidogrel	356 (93,1%)	362 (94,5%)	0,323
Prasugrel	7 (1,8%)	3 (0,7%)	0,201
Ticagrelor	1 (0,2%)	0 (0%)	0,317
Nenhum	7 (1,8%)	8 (2,0%)	0,798
Qualquer ADP^†^	371 (97,1%)	373 (97,3%)	0,820
Terapia Médica a 1 ano, n* (%)	**INSPIRON^**®**^ (374/ 392)**	**Outros SF (375/ 391)**	**“p”**
AAS	357 (95,4%)	355 (94,6%)	0,561
Qualquer inibidor da ADP^†^	337 (90,1%)	332 (88,5%)	0,469

*: Número de stents nos grupos, excluídos óbitos, durante o período mencionado. Qualquer ADP^†^: número de pacientes em uso de qualquer tipo de inibidores de ADP. SF: stent farmacológico; ADP: adenosina difosfato.

O presente estudo tem limitações relacionadas aos estudos não randomizados e à disponibilidade momentânea dos dispositivos. A reestenose pode ter sido subdiagnosticada devido a não realização de cateterismo de rotina, embora a reestenose mais tardia (>12 meses), tende a ser mais relacionada à progressão da doença aterosclerótica à ICP prévia. Ademais, agrupar diferentes DES apenas com base na espessura da haste ou medicamento eluido pode não ser apropriado, uma vez que os outros componentes como o desenho do stent, morfologia das hastes e conexões, presença e tipo do metabolismo de polímeros utilizados, também podem influenciar o desempenho. No entanto, este estudo representa uma significativa amostra de mundo real em centro de alto volume, com critérios de inclusão amplos e dados clínicos altamente aplicáveis.

O desenvolvimento da terapia com balões eluidos de fármacos *(Drug eluted balloons – DEB)* vem sendo testada, com uma proposta racional de evitar o implante de malhas metálicas.^23 , 24 , 44 , 45^ Estudos comparativos sobre implante de stents de última geração *vs.* DEB’s, em lesões “de novo” em pacientes com vasos de muito fino calibre (<2,5mm), como no nosso estudo, são fundamentais para guiar a melhor alternativa terapêutica para estes indivíduos.

## Conclusão

A intervenção coronária percutânea em vasos de muito pequeno calibre com stents farmacológicos contemporâneos demonstram satisfatórios resultados clínicos em 12 meses de seguimento, favoráveis à manutenção dessa terapia e utilização dos stents avaliados.
