# Supplementary material for: Registro de Intervenções Coronárias em Vasos de Muito Pequeno Calibre: Análise de Stent Eluidor de Sirolimus e Outros Stents Contemporâneos
Source: Arq Bras Cardiol. 2026 Feb 27;123(2):e20250145. [Article in Portuguese] doi: 10.36660/abc.20250145 (PMC13128180; doi:10.36660/abc.20250145)
Supplement: Translation

# Translation: Registry of Percutaneous Coronary Interventions in Very Small-Caliber Vessels: Analysis of a Sirolimus-Eluting Stent and Other Contemporary Drug-Eluting Stents

**DOI:** 10.36660/abc.20250145i

**Authors:** João Vitor Slaviero, André Luiz Langer Manica, Rogério Eduardo Gomes Sarmento Leite, Eduarda Paiva Borsa, Marcia Moura Schmidt, Rodrigo Campos Ogando, Julia Kurtz Teixeira, Raphael Boesche Guimarães, Alexandre Damiani Azmus, Carlos A. M. Gottschall

**Affiliations:** 1 Brazil Instituto de Cardiologia, Porto Alegre, RS – Brazil; 2 Brazil Universidade Federal de Ciências da Saúde de Porto Alegre, Porto Alegre, RS – Brazil; 3 Brazil Pontifícia Universidade Católica do Rio Grande do Sul, Porto Alegre, RS – Brazil

**Keywords:** Drug-Eluting Stents, Sirolimus, Percutaneous Coronary Intervention, Coronary Artery Disease

## Abstract

**Background:**

Percutaneous coronary intervention (PCI) in very small-caliber vessels (≤2.25 mm) presents technical challenges and is associated with poorer outcomes compared to larger vessels. The Inspiron® stent, a drug-eluting stent (DES) manufactured in Brazil, has been evaluated in multiple studies; however, data on its performance in vessels smaller than 2.5 mm are lacking.

**Objectives:**

This study aimed to evaluate the clinical outcomes of PCI using DES in very small-caliber vessels and to compare the Inspiron® stent with other contemporary DES platforms. The primary endpoint was major adverse cardiac events (MACE), defined as cardiac death, target lesion myocardial infarction, and clinically driven target lesion revascularization.

**Methods:**

We conducted an observational study including a consecutive sample at a Brazilian reference center from 2017 to 2021. Outcomes were assessed in-hospital, at 30 days, 6 months, and 12 months, using a 5% threshold for statistical significance.

**Results:**

A total of 783 DES were implanted: 47% Inspiron^®^and 46.8% other DES. The mean patient age was 64.7 ± 11 years; 61% were male, and 42% had diabetes mellitus. At 12 months, the overall MACE rate was 4.54% (36/793), with no difference between groups (Inspiron®: 4.6% vs. other DES: 4.6%; p=1.000). Individual endpoints and stent thrombosis rates were also similar (4 [1.0%] vs. 2 [0.5%]; p=0.868).

**Conclusion:**

our findings show that the Inspiron^®^stent has acceptable safety and efficacy and provides comparable 12-month clinical outcomes to other contemporary DES in very small-caliber coronary vessels.

## Introduction

Percutaneous coronary intervention (PCI) has grown increasingly complex due to advances in device technology, operator expertise, and procedural techniques.^1 , 2^Small vessel disease (<2.75 mm) is present in up to 50% of PCI cases.^3 - 5^The advent of 2.0 mm and 2.25 mm stents introduced the term “very small-caliber vessel”.^6^

PCI in small-caliber vessels presents significant challenges and is linked to higher risks of adverse cardiac events, including restenosis and stent thrombosis.^4 , 7 - 16^These vessels often have diffused atherosclerotic disease requiring longer stents, which increases restenosis risk^13^and limits the suitability for surgical revascularization.^14 - 16^

With numerous drug-eluting stent (DES) options available, research focuses on features that predict better outcomes. Thinner stent struts correlate with reduced thrombogenicity and shear stress,^17 - 19^Meta-analyses suggest thinner struts may reduce stent thrombosis, myocardial infarction, and target lesion revascularization (TLR) risks, although these findings lack statistical significance. The type of drug eluted by the stent is another key factor. While DES generally exhibit a class effect in small vessels,^20^Sirolimus-eluting DES have shown better outcomes for TLR and myocardial infarction, particularly in longer lesions.^21^

The Inspiron^®^stent, the first DES manufactured in Brazil, elutes Sirolimus, consists of cobalt-chromium alloy with 75 μm struts, and uses a biodegradable abluminal polymer, making it suitable for very small vessels. Although its safety and efficacy have been evaluated,^17 , 18 , 22^no comparative data exist for vessels ≤2.25 mm *vs.* other contemporary DES.

Given the frequency of PCIs in these vessel sizes and widespread Inspiron^®^use, this study aims to analyze real-world outcomes of PCI with contemporary DES in very small-caliber coronary vessels.

## Methods

### Study design, setting, and population

This investigator-initiated, prospective observational study included a consecutive sample of patients aged over 18 years who were referred to the cardiac catheterization laboratory of a tertiary referral hospital in southern Brazil between 2017 and 2021. All patients underwent PCI of very small-caliber vessels using either the INSPIRON^®^stent or other contemporary DES. The indication for PCI was determined by the attending medical team for each patient. Patients with stents implanted in coronary lesions of larger calibers (≥2.5 mm), as well as those undergoing PCI without DES implantation, were excluded from this study.

Clinical data were collected from medical records, outpatient follow-up visits, and telephone contacts. Clinical outcomes were assessed during hospitalization and at 30 days, 6 months, and 12 months post-PCI. Repeat coronary angiography was performed only in patients with a clinical indication for invasive reassessment as determined by the attending physicians; routine angiographic or functional testing was not performed during follow-up.

The study protocol was approved by the Institutional Research and Ethics Committee of the hospital and was conducted in accordance with Resolution CNS 466/2012.

### Procedural aspects

All patients received dual antiplatelet therapy (DAPT) either before or immediately after the PCI procedure, and unfractionated heparin was administered during the intervention. The procedural strategy, including the choice and duration of DAPT, was determined by the attending medical team. Except in cases guided by intravascular imaging, the reference vessel diameter was estimated visually during angiography.

### Stents

All patients received at least one *INSPIRON* ^*®*^or other DES. The choice of stent type was based on operator discretion and device availability at the center. The characteristics of the stents used are shown in Table 1 .

**Table 1:** – Drug-eluting stents used

Stent	N (%)	Material	Strut Thickness	Drug	Polymer
**Inspiron** ^**®**^	392 (50.1%)	Cobalt-Chromium	75μm	Sirolimus	Biodegradable
**Resolute Onyx/ Integrity** ^**®**^	218 (27.8%)	Cobalt-Chromium	81/91μm	Zotarolimus	Durable
**Promus** ^**®**^	73 (9.7%)	Platinum-Chromium	81μm	Everolimus	Biodegradable
**Xience** ^**®**^	37 (4.7%)	Cobalt-Chromium	81μm	Everolimus	Durable
**Synergy** ^**®**^	30 (3.8%)	Platinum-Chromium	74μm	Everolimus	Biodegradable
**Orsiro** ^**®**^	19 (2.4%)	Cobalt-Chromium	60μm	Sirolimus	Biodegradable
**Ultimaster** ^**®**^	8 (1.0%)	Cobalt-Chromium	80μm	Sirolimus	Biodegradable
**Supraflex** ^**®**^	6 (0.8%)	Cobalt-Chromium	60	Sirolimus	Biodegradable

Drug-eluting stents analyzed in the sample, presented in descending order of frequency, included the metallic scaffold material, strut thickness, eluted drug, and polymer type.

### Study endpoints

The primary endpoint was *Major Adverse Cardiovascular Events (MACE)* , a composite of Cardiac Death, Target Lesion Myocardial Infarction, and Clinically Driven TLR. In addition, the individual components of the primary endpoint were analyzed, as well as the endpoint of Stent Thrombosis, defined as acute myocardial infarction associated with occlusion of a previously implanted 2.00 mm or 2.25 mm DES, classified as *definite or confirmed stent thrombosis.* ^20^

The main study objective was to evaluate the overall composite endpoint in the sample and compare outcomes between *INSPIRON* ^*®*^and other contemporary DES available at the center over a one-year follow-up period. Differences in MACE rates were compared according to strut thickness (<80 µm vs. >80 µm) and the type of drug eluted (Sirolimus vs. other agents).

Angiographic success of PCI was defined as residual stenosis <20% with TIMI 3 flow, without side-branch occlusion, flow-limiting dissection, distal embolization, or angiographic thrombus.^21^

### Statistical analysis

Data were analyzed using the IBM *Statistical Package for the Social Sciences* (SPSS), version 20.0. Categorical variables are presented as absolute numbers (n) and percentages (%), and continuous variables as mean ± standard deviation. The normality of distribution for continuous variables was assessed using the Kolmogorov–Smirnov test. Associations between categorical variables were tested using the Chi-square test, and differences in continuous variables were assessed with the unpaired Student’s *t* -test. A two-tailed p-value <0.05 was considered statistically significant. Survival curves were estimated using the Kaplan–Meier method, and group differences were compared using the log-rank test, adopting a 5% significance level.

### Study power

Given the available sample size of 391 and 392 patients per group, the study had over 80% power to detect differences of approximately six percentage points. Power calculations were performed using the WINPEPI software, version 11.65.

## Results

From January 2017 to December 2021, a total of 834 stents were implanted and included in the database. Of these, 783 (93.8%) stents met the inclusion criteria, were successfully implanted, and had at least 12 months of follow-up: 392 *INSPIRON* ^*®*^stents (47%) and 391 “Other DES” (46.8%). These 834 stents were implanted in 762 patients (1.03 stent per patient). Seven stents (0.8%) were excluded from follow-up (four in the *INSPIRON* ^*®*^group and three in the “Other DES” group, 1.02% vs. 0.76%, p=0.760) due to four cases of no-reflow, two cases of stent delivery failure due to severe proximal tortuosity and calcification, and one death caused by ventricular fibrillation during stent deployment ( Figure 1 ).

**Figure 1:** – Flowchart.

The rates of hypertension, diabetes mellitus, active smoking, and dyslipidemia were similar between groups. Mean age was slightly higher in the “Other DES” group, reaching statistical significance, though the small numeric difference had no clinical impact on outcomes. Prior heart failure was more frequent in the *INSPIRON* ^*®*^group, while acute coronary syndrome (ACS) accounted for 74.7% of the PCI indications, with unstable angina being the most common diagnosis (40.4%), significantly higher in the “Other DES” group. Other clinical characteristics are shown in Table 2 .

**Table 2:** – Clinical and Demographic Characteristics

Clinical and Demographic Characteristics	Stents (n = 783)	INSPIRON^**®**^(n = 392; 50%*)	Other DES (n = 391; 50%*)	p
Age (years)	64.7	63.3 ±10.98	66.13±11.15	0.001
BMI (kg/m^2^)	27.5	27.26 ±3.55	27.64 ±3.73	0.138
Male sex	480(61.3%)	227(47.2%)	253(52.8%)	0.060
Serum creatinine (mg/dL)	1.04	1.02 ±0.38	1.06±0.40	0.249
Hypertension	603(77%)	302 (50%)	301 (50%)	1.000
Family history of CAD	69 (8.8%)	37 (59.6%)	32 (46.4%)	0.622
Current smoker	293(37.4%)	156 (53.3%)	137 (46.7%)	0.193
Diabetes mellitus	329 (42%)	177(53.7%)	152 (46.3%)	0.088
Insulin-treated diabetes	92 (11.7%)	55 (59.7%)	37(40.3%)	0.091
Dyslipidemia	259(33.1%)	121 (46.7%)	138 (53.3%)	0.105
Heart failure (LVEF <50%)	48 (6.1%)	33 (68.7%)	15 (31.3%)	0.012
Previous stroke	27 (3.4%)	15 (55.5%)	12 (44.5%)	0.700
Previous myocardial infarction	121(15.5%)	64 (52.8%)	57(47.2%)	0.563
Previous PCI	162(20.7%)	73 (45%)	89 (55%)	0.180
Previous CABG	64 (8.2%)	34 (53.2%)	30 (46.8%)	0.703

Proportions refer to variables analyzed separately and not to the total sample size. DES: drug-eluting stents; BMI: body mass index; HTN: hypertension; CAD: coronary artery disease; DM: diabetes mellitus; HF-LVEF <50%: heart failure with left ventricular ejection fraction <50%; CVA: cerebrovascular accident; PCI: percutaneous coronary intervention; CABG: coronary artery bypass grafting.

*INSPIRON* ^*®*^stents were more frequently implanted in the circumflex artery, right coronary artery, and posterior descending artery compared with the “Other DES” group. There were no significant differences between groups regarding stent length, access route, or percentage of pre-dilated lesions. PCIs using *INSPIRON* ^*®*^stents showed lower rates of rotational atherectomy and intravascular ultrasound (IVUS) use, though absolute rates of these devices were very low, preventing correlation with events. Additional angiographic and procedural data are presented in Table 3 .

**Table 3:** – Angiographic and procedural characteristics

Variable	Total (n = 783)	INSPIRON^**®**^(n = 392; 50%*)	Other DES (n = 391; 50%*)	p
**Target coronary vessel**				
Left anterior descending artery	293 (36%)	157 (53.5%)	136 (46.4%)	0.127
Left circumflex artery	139 (18%)	87 (62.5%)	52 (37.4%)	**0.001**
Right coronary artery	171 (22%)	102 (59.6%)	69 (40.3%)	**0.004**
Diagonal branch	68 (8.6%)	27 (39.7%)	33 (60.2%)	0.420
Obtuse marginal branch	70 (8.9 %)	41 (58.5%)	29 (41.4%)	0.132
Posterior descending artery	31 (3.9%)	22 (70.9%)	8 (29%)	**0.009**
Posterolateral branch	14 (1.7%)	8 (57.1%)	6 (42.8%)	0.546
Venous graft	7 (0.89%)	1 (14.3%)	6 (85.7%)	0.057
**Clinical presentation**				
ST-elevation myocardial infarction (STEMI)	145(18.5%)	81 (55.8%)	64 (44.1%)	0.117
Non-ST-elevation myocardial infarction (NSTEMI)	124(15.8%)	74 (59.6%)	50 (40.3%)	0.180
Unstable angina	316(40.4%)	127 (40.1%)	189 (59.8%)	**0.001**
Stable angina	198(25.3%)	110 (55.5%)	88 (44.4%)	0.070
**Functional assessment**				
Invasive physiology (FFR/iFR)	7 (0.89%)	1 (14.2%)	6 (85.7%)	0.057
Myocardial scintigraphy	18 (2.3%)	6 (33.3%)	12 (66.6%)	0.152
Stress echocardiography	1 (0.1%)	1 (100%)	0 (0%)	0.320
Exercise treadmill test	11 (1.4%)	5 (45.4%)	6 (54.5%)	0.860
**Procedural aspects**				
Predilation	710(90%)	355 (50%)	355 (50%)	0.981
Thrombus aspiration	6 (0.8%)	4 (66.6%)	2 (33.3%)	0.412
Rotational atherectomy	9 (1.1%)	0 (0.0%)	9 (100%)	**0.002**
Intravascular ultrasound (IVUS)	8 (1.0%)	0 (0.0%)	8 (100%)	**0.004**
Postdilation	369(47.1%)	171 (46.3%)	198 (53.6%)	**0.049**
Radial arterial access	588(75.1%)	285 (48.4%)	303 (51.5%)	0.121
Femoral arterial access	195(24.9%)	98 (50.2%)	97 (49.7%)	0.950
Mean stent length (mm)	21.46	22.05±6.0mm	21.45±7.2mm	0.717
**Stent diameter**				
- 2.0 mm	16 (2.0%)	4 (25%)	12 (75%)	0.068
- 2.25 mm	767 (97.9%)	386 (50.3%)	381 (49.6%)	0.862

*Proportions refer to variables analyzed separately and not to the total sample size. DES: drug-eluting stent; IVUS: intravascular ultrasound.

The overall MACE rate was 4.54% (36/793) and statistically equivalent between the compared groups ( Table 4 ): 18 events in the INSPIRON® group and 18 in the “Other DES” group ( Figure 2 ), as well as in each individual endpoint. he incidence of stent thrombosis was similar between stent types throughout all evaluated periods ( Figure 3 ).

**Table 4:** – Clinical Outcomes at 12-Month Follow-Up

Períod Group / Number of Stents	In-Hospital		30 days		6 months		1 year	
	Inspiron^**®**^(n=392)	Other DES (n=391)	Inspiron^**®**^(n=392)	Other DES (n=391)	Inspiron^**®**^(n=392)	Other DES (n=391)	Inspiron^**®**^(n=392)	Other DES (n=391)
**MACE**	0 (0%)	1(0.3%)	6(1.5%)	3 (0.8%)	10 (2.5%)	7(1.8%)	18(4.6%)	18(4.6%)
**p**	**p = 0.499**		**p = 0.505**		**p = 0.625**		**p = 1.000**	
**Target Lesion Revascularization**	0 (0%)	0 (0%)	1 (0.2%)	0 (0%)	2 (0.5%)	0 (0%)	4 (1.0%)	7 (1.8%)
**p**	**p = 1.000**		**p = 0.999**		**p = 0.499**		**p = 0.384**	
**Target Lesion Myocardial Infarction**	0 (0%)	1 (0.3%)	2(0.5%)	2(0.5%)	2(0.5%)	2(0.5%)	5(1.3%)	3(0.8%)
**p**	**p = 0.499**		**p = 1.000**		**p = 1.000**		**p = 0.725**	
Cardiac Mortality*	0 (0%)	0(0%)	3 (0.8%)	1 (0.3%)	6 (1.5%)	5(1.3%)	9(2.3%)	8(2.1%)
**p**	**p = 1.000**		**p = 0.624**		**p = 0.999**		**p = 0.999**	

MACE: Major Adverse Cardiovascular Events (composite of cardiac death, target lesion myocardial infarction, and clinically driven target lesion revascularization); TLR: Target Lesion Revascularization (clinically driven); Cardiac mortality: includes confirmed and probable cardiac deaths.

**Figure 2:** – MACE Events. Cumulative number of MACE (Major Adverse Cardiovascular Events — cardiac mortality, target lesion myocardial infarction, and clinically driven target lesion revascularization) during the follow-up periods for each stent group.

**Figure 3:** – Stent Thrombosis During the Evaluated Periods. Cumulative number of stent thrombosis events during the follow-up periods for each stent group.

The confirmed and probable cardiac mortality rate in the overall cohort was 2.14% (17/793), similar between study groups. Clinical follow-up was comparable across groups, with a loss to follow-up of 42 patients (5.5%) over one year.

During a one-year follow-up, event-free survival curves were similar ( Figure 4 ). At 6- and 12-month follow-up, both groups maintained comparable DAPT use ( Table 6 )..

**Figure 4:** – Kaplan-Meier Curve. Kaplan-Meier cumulative survival curve free from MACE (Major Adverse Cardiovascular Events — cardiac mortality, target lesion myocardial infarction, and clinically driven target lesion revascularization) during the 365-day follow-up period.

**Table 6:** – Antiplatelet Medical Therapy

*Antiplatelet Therapy at 6 Months, n (%) **	INSPIRON^**®**^(382/ 392)	Other DES (383/ 391)	p
Aspirin	364 (95.2%)	369 (96.3%)	0.242
**ADP Inhibitors**			
Clopidogrel	356 (93.1%)	362 (94.5%)	0.323
Prasugrel	7 (1.8%)	3 (0.7%)	0.201
Ticagrelor	1 (0.2%)	0 (0%)	0.317
None	7 (1.8%)	8 (2.0%)	0.798
Any ADP inhibitor^†^	371 (97.1%)	373 (97.3%)	0.820
*Antiplatelet Therapy at 1 Year, n (%) **	**INSPIRON^**®**^(374/ 392)**	**Other DES (375/ 391)**	**p**
Aspirin	357 (95.4%)	355 (94.6%)	0.561
Any ADP nhibitor^†^	337 (90.1%)	332 (88.5%)	0.469

*: Number of stents in each group, excluding deaths during the corresponding follow-up period.^†^Number of patients receiving any ADP inhibitor (Clopidogrel, Prasugrel, or Ticagrelor). ADP: adenosine diphosphate; DES: drug-eluting stent.

The incidence of stent thrombosis was similar between stent types throughout all evaluated periods ( Table 5 ). At 6- and 12-month follow-up, both groups maintained comparable DAPT use ( Table 6 ).

**Table 5:** – Stent Thrombosis

Period Type of Stent, n (%)	Intra-hospitalar		30 days		6 months		1 year	
	Inspiron^**®**^	Others	Inspiron^**®**^	Others	Inspiron^**®**^	Others	Inspiron^**®**^	Others
Stent thrombosis*	0 (0%)	1 (0.3%)	2 (0.5%)	2(0.5%)	2(0.5%)	2 (0.5%)	4(1.0%)	2(0.5%)
**p**	**p = 0.499**		**p = 1.000**		**p = 1.000**		**p = 0.868**	

Cumulative rate of stent thrombosis over follow-up periods. *Stent thrombosis: confirmed stent thrombosis.

## Discussion

This contemporary real-world registry, which evaluated Brazilian clinical data from patients undergoing PCI with stent implantation in very small-caliber vessels, demonstrated low MACE rates and comparable outcomes among the different available stent platforms ( Central Illustration ), consistent with data from previous literature.^23 , 24^

The presence of diabetes mellitus is often associated with diffuse atherosclerotic coronary disease in small vessels and is considered a predictor of restenosis and stent thrombosis after PCI with DES.^25 - 35^In our sample, among the stents associated with MACE, 42% (15/36) were implanted in patients with known diabetes mellitus — the same proportion as in the overall cohort. Most patients undergoing PCI required stents longer than 20 mm; however, the mean stent length was not greater among those associated with MACE compared with event-free stents (21.98 mm vs. 21.43 mm, p=0.820). Although certain clinical characteristics differed between groups, analysis of MACE for *INSPIRON® vs.* “Other DES” showed no significant difference among patients with heart failure and reduced ejection fraction (<50%) (4/18 [22%] vs. 3/18 [17%], p=0.67) or those presenting with acute coronary syndromes (8/18 [44.4%] vs. 9/18 [50%], p=0.74). The small number of events during follow-up precluded multivariate analysis of other clinical and procedural variables.

The potential benefit of thinner stent struts, which may cause less endothelial injury, was evaluated in our cohort when comparing DES with <80 µm *vs.* >80 µm strut thickness. Although no statistical significance was found between *INSPIRON®* and other DES (48% vs. 52%, p=1.010), it is plausible that a larger sample or studies including routine angiographic follow-up might detect differences, as the occurrence of acute or chronic ischemic symptoms — the main indication for invasive re-evaluation — may have been underestimated due to the small myocardial territory at risk.

When analyzing results by eluted drug, our data demonstrate statistical equivalence in MACE between Sirolimus-eluting stents ( *INSPIRON®* ) and devices eluting other agents, suggesting a class effect among currently available DES, in line with existing evidence.^36 - 39^These findings also indicate that engineering and biochemical characteristics of contemporary DES platforms may mitigate the relative importance of the specific drug used.

The use of intravascular imaging (particularly IVUS) improves acute and long-term angiographic and clinical outcomes.^40 - 43^However, its use in our sample was very limited — only 1% (8/783) of cases — since most procedures were performed in public health system patients, where this technology is not routinely funded. Moreover, IVUS use in vessels <2.25 mm can be technically challenging and may increase the risk of iatrogenic plaque injury,^41^which might have influenced operator decisions.

The diffuse distribution pattern of stent thrombosis events observed in our registry suggests that acute procedural factors such as stent underexpansion or significant coronary dissection were not major contributors to adverse outcomes Table 5 .

DAPT use and duration were similar between groups throughout follow-up, with a high maintenance rate of DAPT at 12 months, which may have influenced the low event frequency ( Table 6 ).

This study has limitations inherent to nonrandomized designs and device availability at the time of PCI. Restenosis might have been underdiagnosed due to the absence of routine angiographic follow-up; however, late restenosis (>12 months) is usually more related to atherosclerotic progression than to the previous PCI. Grouping different DES solely by strut thickness or drug elution may not be entirely appropriate, as other design factors — such as strut geometry, connection morphology, and polymer degradation — may also influence outcomes. Nevertheless, this study represents a significant real-world sample from a high-volume tertiary center, with broad inclusion criteria and clinically relevant findings.

The emergence of drug-eluting balloons (DEB) has introduced a rational alternative for avoiding metallic scaffolds.^23 , 24 , 44 , 45^Comparative studies between new-generation DES and DEB in *de novo* lesions of very small-caliber vessels (<2.5 mm), similar to those in our study, are essential to determine the optimal therapeutic approach for these patients.

## Conclusion

PCI in very small-caliber vessels using contemporary DES demonstrates satisfactory clinical outcomes at 12-month follow-up, supporting the continued use of this therapeutic strategy and of the evaluated stent platforms.
